# Marine Biofilm Bacteria Evade Eukaryotic Predation by Targeted Chemical Defense

**DOI:** 10.1371/journal.pone.0002744

**Published:** 2008-07-23

**Authors:** Carsten Matz, Jeremy S. Webb, Peter J. Schupp, Shui Yen Phang, Anahit Penesyan, Suhelen Egan, Peter Steinberg, Staffan Kjelleberg

**Affiliations:** 1 School of Biotechnology and Biomolecular Sciences and Centre for Marine Bio-Innovation, University of New South Wales, Sydney, Australia; 2 Division of Cell and Immune Biology, Helmholtz Centre for Infection Research, Braunschweig, Germany; 3 School of Biological Sciences, University of Southampton, Southampton, United Kingdom; 4 Marine Laboratory, University of Guam, Mangilao, Guam, United States of America; 5 School of Biological, Earth and Environmental Sciences and Centre for Marine Bio-Innovation, University of New South Wales, Sydney, Australia; Monterey Bay Aquarium Research Institute, United States of America

## Abstract

Many plants and animals are defended from predation or herbivory by inhibitory secondary metabolites, which in the marine environment are very common among sessile organisms. Among bacteria, where there is the greatest metabolic potential, little is known about chemical defenses against bacterivorous consumers. An emerging hypothesis is that sessile bacterial communities organized as biofilms serve as bacterial refuge from predation. By testing growth and survival of two common bacterivorous nanoflagellates, we find evidence that chemically mediated resistance against protozoan predators is common among biofilm populations in a diverse set of marine bacteria. Using bioassay-guided chemical and genetic analysis, we identified one of the most effective antiprotozoal compounds as violacein, an alkaloid that we demonstrate is produced predominately within biofilm cells. Nanomolar concentrations of violacein inhibit protozoan feeding by inducing a conserved eukaryotic cell death program. Such biofilm-specific chemical defenses could contribute to the successful persistence of biofilm bacteria in various environments and provide the ecological and evolutionary context for a number of eukaryote-targeting bacterial metabolites.

## Introduction

Predators are potent agents of mortality and natural selection in biological communities. Plants and animals synthesize a broad range of secondary metabolites that are deterrent or toxic to their consumers, thus functioning as defense compounds. Such chemicals are often common in sessile eukaryotic organisms such as marine sponges and corals, seaweeds and terrestrial plants [1]–[4], which lack escape or avoidance mechanisms. However, chemically-mediated antipredator defenses of bacteria and their ecological and evolutionary consequences remain a greatly understudied field. Particularly, the increasing number of biologically active compounds isolated from marine bacteria raises the question of their ecological functions [5].

In many aquatic ecosystems, a common mode of life for bacteria is in biofilms, i.e. sessile high-density consortia of cells glued together by an exopolymeric matrix. Whilst bacteria in the plankton typically occur at concentrations of 10^5^ to 10^7^ cells ml^−1^, cell densities in bacterial biofilms tend to be two or three orders of magnitude higher. The ability of biofilm bacteria to achieve high densities has been ascribed to various factors, including greater resistance and/or tolerance to exogenous stresses or the accumulation of nutrients at surfaces [6], [7]. However, an alternative hypothesis for the prevalence of biofilms is that they serve as a protective niche against predation [8], [9], allowing for higher densities of cells than in planktonic environments.

Predation by phagotrophic protists, the protozoa, constitutes a major mortality factor for both planktonic and biofilm bacteria [10]–[12]. Aggregations of bacterial cells are rapidly detected and grazed by protozoa [13], [14], which in turn can drive shifts in bacterial communities from edible to grazing-resistant species or ecotypes [15]. In the plankton, size-selective protozoan grazing typically favors less edible ultramicrobacteria (cell volume <0.1 µm^3^) or elongated, filamentous cells [16]–[18]. Bacterial swimming speeds of >30 µm s^−1^, particularly in combination with small cell size, can also significantly reduce prey capture and handling probabilities of bacterivorous flagellates [19].

Although bacteria-protist interactions in biofilms remain largely unexplored, bacterial morphotypes found in grazing-exposed freshwater biofilms do not appear to be comprised of the grazing-resistant phenotypes reported for bacterioplankton [12]. Escape by swimming by biofilm bacteria would eliminate the biofilm, and the formation of filamentous, less edible cell morphologies may be limited by strong competition for nutrients and space at the typically high cell densities found in biofilms. Thus, by analogy to sessile macroorganisms, we might expect that chemical defenses would be an important mode of defense against predators in the high cell density, clonal consortia that comprise biofilms. As a corollary, we might further expect – as suggested by the existing literature - that planktonic populations would rely more heavily on alternative defenses such as cell morphology or escape, with chemical defenses less prevalent (though we note the increasing evidence for chemical defenses in marine eukaryotic plankton [20]).

In this study, we for the first time compared the presence and efficacy of chemical defenses in biofilms vs. planktonic populations of marine bacteria. We found a much greater prevalence of chemical defenses in the biofilm mode of life. We then went on to investigate the nature and mode of action of one such defense which is highly effective against a wide range of ecologically important protozoan predators. By contrasting the occurrence of chemical defense in biofilm and plankton populations, we tested the potential adaptive advantage for bacteria growing as biofilms when exposed to protozoan grazers.

## Results and Discussion

Our first hypothesis, that evidence for chemical defenses would be more prevalent in biofilm vs. planktonic populations of bacteria, was tested using members of the well-characterized biofilm community found on the surface of the marine macroalga *Ulva australis* [21]–[25]. Clone libraries of the 16S ribosomal RNA gene have previously uncovered considerable bacterial diversity in this community, including representatives of the five main bacterial groups, Bacteriodetes, Planctomycetes, α-, δ-, and γ-Proteobacteria [25]. Besides some host-specific taxa [25], the culturable fraction of the bacterial community associated with *U. australis* is comprised of bacterial species found also in the planktonic community [21]–[23]. A diverse set of bacterial strains from this community were screened for their effect on protozoan predators when the bacteria were grown either as a biofilm or planktonically. Protozoa generally show a strict niche separation into suspension and surface feeding species, thus lacking species that are capable of efficiently utilizing both suspended and attached prey. To test for biofilm versus plankton effects, we used an experimental system contrasting two niche-specific predatory flagellates which are typically found in coastal ecosystems [26]. The surface feeder *Rhynchomonas nasuta* and the suspension feeder *Cafeteria roenbergensis* have repeatedly been isolated from coastal microbial communities and are among the 20 most commonly reported species of marine heterotrophic flagellates [27]. Despite their contrasting feeding modes, these two predators show high similarity in cellular features that are relevant for the comparison of feeding experiments, e.g. cell size, feeding rates and susceptibility to bacteria producing biologically active compounds [26], [28].

**Figure 1:**
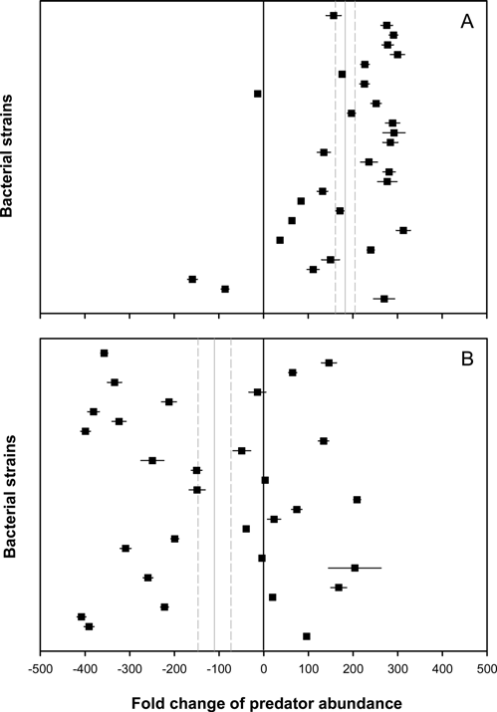
Grazing resistance and antiprotozoal activity in biofilm bacteria. Change in relative abundance of the protozoan predators *C. roenbergensis* feeding on planktonic populations (A) and *R. nasuta* feeding on biofilm populations (B) of bacteria isolated from the marine green alga *U. australis*. Bacteria were considered to be edible or toxic if predator numbers showed a significant increase or decrease relative to the non-food control treatment. Data are means±SD of four replicates. Dashed lines represent total means±SE of 30 strains.

Our screen revealed that planktonic populations of the 30 bacterial strains tested were widely edible, as evidenced by a moderate to high increase in cell numbers of the suspension feeder *C. roenbergensis* (180-fold increase on average, Fig. 1A). In 27 bacterial strains (90%), we found a significant increase in predator numbers relative to the non-food control treatment (P<0.01). Cell yield values, a measure describing the number of bacteria consumed to produce one flagellate cell, were typically 10 to 30 bacteria flagellate^−1^, indicating high food quality for planktonic cells of 22 strains (73%). Grazing of *C. roenbergensis* resulted in a significant reduction of planktonic cell numbers of all 30 bacterial strains relative to the predator-free control (P<0.001, see Supporting information, Fig. S1A).

In contrast, the majority of biofilm populations were resistant or toxic to the surface feeder *R. nasuta* (Fig. 1B). Biofilms of 17 strains (57%) caused a significant reduction in predator numbers relative to the non-food control treatment (P<0.001), which concurred with relatively stable biofilm biomasses (see Supporting information, Fig. S1B). Only eleven strains were significantly reduced by *R. nasuta* (P<0.01). Numbers of *R. nasuta* remained unchanged in biofilms of five strains, while eight bacterial strains supported growth of the flagellate predator (P<0.01). Nineteen out of the 22 strains that were resistant or toxic to the surface feeder (when grown as biofilms) showed at the same time high susceptibility to the suspension feeding flagellate (when grown planktonically). Biofilms have recently been proposed to serve as refuge from protozoan grazing for bacterial pathogens such as *Vibrio cholerae* [26]. The current dataset expands this concept to non-pathogenic bacteria and across a broader range of bacterial taxa, suggesting that biofilm-specific resistance against predation is more widespread than previously thought.

Our previous studies have shown that the formation of less edible biofilm structures, such as microcolonies and exopolymers, may slow down flagellate growth but still leads to a several hundredfold increase in flagellate numbers [26], [29], [30]. Moreover, constant flagellate numbers in non-food control treatments of our present study indicate that the reduction in predator numbers was not a result of starvation. Thus, the substantial decline of protozoan numbers strongly suggests that grazing resistance was not based on the physical inaccessibility of biofilm cells, or poor nutritive value, but rather chemical interference with predator viability. To confirm this for a more restricted set of bacteria, we investigated in more detail biofilms of two γ-proteobacteria, *Pseudoalteromonas tunicata* and *Microbulbifer* sp., which showed the strongest negative effects against the flagellate consumers.

**Figure 2:**
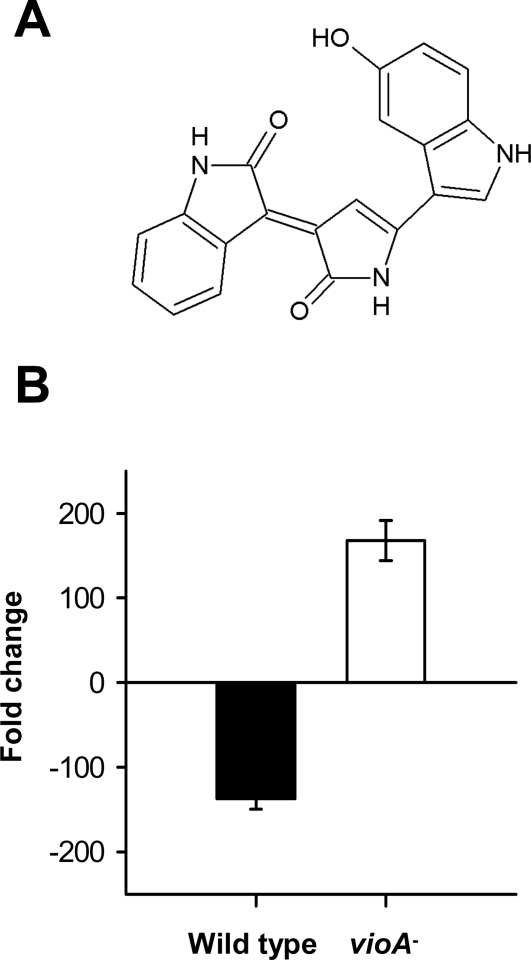
Defensive metabolite violacein. (A) The molecular structure of violacein, the antiprotozoal constituent of *P. tunicata* cells. (B) Mortality and growth of *R. nasuta* on biofilms of wild-type *P. tunicata* and the violacein-deficient *vioA* mutant. Data are means±SD of four replicates.

To identify the putative protozoa-active compound produced by *P. tunicata*, we chose a dual approach of chemical extraction and transposon mutagenesis. Antiprotozoal activity of biofilm extracts was evaluated by monitoring the total number and the number of active flagellate cells in cultures of *C. roenbergensis* and *R. nasuta* growing on heat-killed *P. aeruginosa*. Bioassay-guided chromatographic fractionation of crude biofilm material followed by NMR-spectroscopic analysis revealed that the antiprotozoal activity of *P. tunicata* is elicited by the purple pigment violacein, an L-tryptophan-derived alkaloid consisting of three structural units: 5-hydroxyindole, 2-pyrrolidone and oxindole (Fig. 2A and see Supporting information, Table S1). Antiprotozoal activity was also detected for the violacein derivative deoxyviolacein, which is produced at 3-fold lower quantities. Violacein was first described from cellular extracts of the β-proteobacterium *Chromobacterium violaceum* [31]. Analysis of the *P. tunicata* genome sequence revealed a 8 kb region encoding a putative biosynthetic gene cluster consisting of five open reading frames (Genbank accession numbers ZP01133841 to ZP0113384), each with high (>40%) predicted amino acid sequence identity to the violacein operon *vioABCDE* of *C. violaceum* [32], [33].

The role of violacein in the killing of protozoan predators by *P. tunicata* was supported through the analysis of a *vioA* mutant, defective in violacein biosynthesis. While biofilms of the *P. tunicata* wild type killed the flagellate *R. nasuta* within 24 hours, the *vioA* mutant was completely grazed resulting in significantly higher predator numbers compared to the wild type and the non-food control treatment (P<0.001, Fig. 2B). Similar effects of *P. tunicata* wild type and violacein-negative mutant as well as of purified violacein were found against a broad spectrum of protozoan predators, including amoebae, ciliates, and flagellates (see Supporting information, Table S2). PCR-amplification of the putative *vioA* gene and biochemical analysis of cell extracts revealed that violacein is synthesized by another two species found in *U. australis* biofilms, *Microbulbifer* sp. and *Pseudoalteromonas ulvae*. Comparing the isolated strains with the known violacein-producers *C. violaceum, Janthinobacterium lividum* and *Pseudoalteromonas luteoviolacea* revealed that, despite spanning several genera, all 6 species share a niche preference for sessile microbial communities, such as those found in biofilms, sediments and soils [21], [34]–[36]. The putative biofilm prevalence of violacein-producers may find support in the fact that the violacein gene cluster has not been detected in the currently largest metagenomics sequence database of pelagic ocean waters [37], [38].

**Figure 3:**
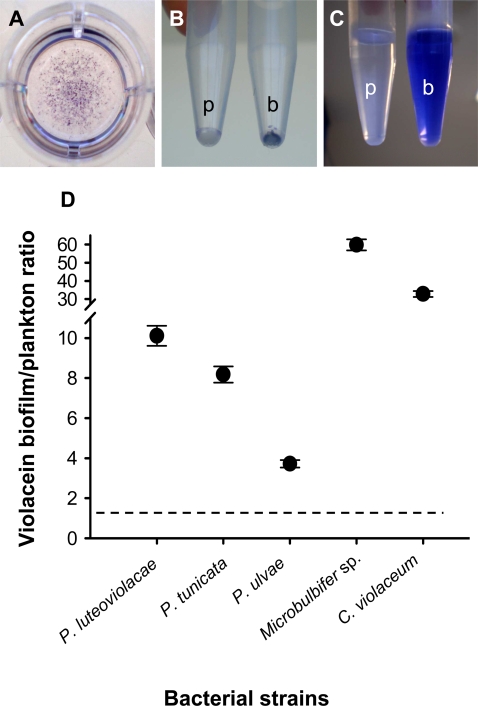
Biofilm-enhanced production of violacein. (A) Formation of violacein-containing biofilms by *Microbulbifer* sp. at well bottom. (B) Cell pellets of planktonic (p) and biofilm (b) bacteria. (C) Butanol extracts from planktonic (p) and biofilm (b) bacteria. (D) Relative violacein content of biofilm and planktonic populations of five bacterial species. Dashed line indicates an equal distribution. Data are means±SD of four replicates.

To test whether violacein is produced predominantly by biofilm populations, we analyzed five violacein-producing bacteria, *P. tunicata*, *P. ulvae*, *P. luteoviolacea*, *Microbulbifer* sp. and *C. violaceum*, for their violacein biosynthesis rates in biofilms versus planktonic cells. While all five species grew to considerable cell densities in plankton (≥2×10^7^ cells ml^−1^) and biofilm (OD_490 nm_≥0.2), cell extracts from biofilms contained 3 (for *P. ulvae*) to 59 (for *Microbulbifer* sp.) times more violacein per protein biomass than the corresponding plankton extracts (Fig. 3). Differences between biofilm and plankton extracts were significant for all five species (P<0.001).

In nutrient addition experiments, we examined the influence of cell density on the relative violacein content in biofilms versus planktonic bacteria. Although population densities of both biofilm and plankton were positively correlated with the nutrient concentrations applied, the violacein production by planktonic cells remained minimal at low to intermediate nutrient levels while the violacein content in biofilm populations was relatively high and increased continually. However, the relative differences in violacein production between biofilm and planktonic bacteria became significantly smaller with increasing cell densities at high nutrient concentrations (P<0.01, see Supporting information, Fig. S2). It appears that the localized high cell densities in biofilms allow for higher violacein content particularly in environments of low productivity. The central role of population density for the biofilm-enhanced production of violacein is intriguing, given that violacein biosynthesis in *C. violaceum* is regulated by quorum sensing [39]. Assuming biofilm and cell density dependent gene regulation for the five violacein-producing species, our data may illustrate the adaptive advantage of clonal growth and cellular cooperation as found in biofilms for the synthesis of antipredator compounds. As with quorum sensing compounds themselves, it may only be adaptive for bacteria to produce violacein when they are in sufficiently high densities (e.g., in biofilms) to produce concentrations that are active (in this case, inhibitory against predators).

To further investigate the effectiveness of violacein-mediated defense, we analyzed concentration-dependent effects on feeding, growth and viability of the flagellate *R. nasuta*, the ciliate *Tetrahymena* sp. and the amoeba *Acanthamoeba castellanii*. In these experiments, we tested (i) mono-species biofilms of *P. tunicata*, *P. ulvae*, *P. luteoviolacea*, and *Microbulbifer* sp. and (ii) purified violacein at concentrations from 50 to 0.1 µM. Although biofilms were grown under low nutrient conditions, all three protozoan predators showed 100% mortality within 24 hours when feeding on biofilms of all four violacein-producing species (see Supporting information, Table S3). Chemical extractions from cell pellets and cell-free culture supernatants revealed that violacein is stored intracellularly. Sub-cellular fractionation of *P. tunicata* cells further suggest that violacein is associated with the outer membrane and accumulates in the periplasm. It is possible that periplasmic storage aids to increase the defense efficiency by minimizing the loss of compounds into seawater, avoiding autotoxicity and ensuring direct contact with a potential consumer. Our findings of cell bound storage may also implicate a primary ecological function of violacein in antipredator defense rather than in competitor inhibition [40].

**Figure 4:**
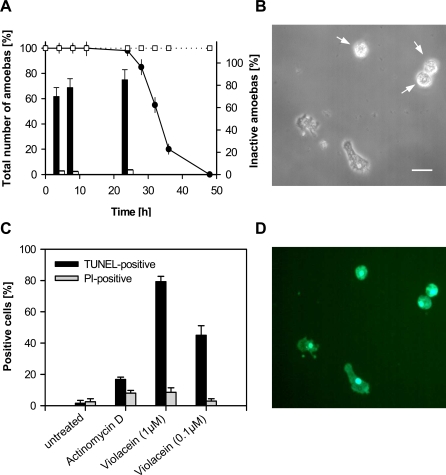
Induction of predator death program. (A and B) Inactivation and (C and D) apoptosis-like changes in the amoeba *A. castellanii* upon exposure to violacein. (A) Relative number of total amoebae and inactive cells in the absence (open symbols) or presence of 1 µM violacein (filled symbols). (B) Rounded cell morphology indicates inactive/stressed amoeba. Scale bar, 20 µm. (C) DNA degradation and maintenance of membrane integrity in *A. castellanii* after 24 hours as revealed by TUNEL and propidium iodide (PI) staining. Positive control is treatment with actinomycin D. (D) Bright fluorescence indicates fragmentation of nuclear DNA in *A. castellanii* upon 24-hours-exposure to 1 µM violacein. Data are means±SD.

When exposed to micro- and submicromolar concentrations of pure violacein, protozoan cells showed subtle morphological changes that are indicative of stress. While cell numbers of *A. castellanii*, for example, remained stable over 24 hours, up to 85% of cells exhibited the inactive rounded cell type (Fig. 4A and B). This state of feeding inactivity was followed by a sharp decline of *A. castellanii* cell numbers about 26 hours after the onset of feeding inhibition. Within hours 100% of *A. castellanii* cells were lysed in the presence of 1 µM violacein, but not in stationary phase cultures of *A. castellanii* without violacein exposure (Fig. 4A). Similar to the killing of planktonic flagellates by *C. violaceum* [41], microscopic analysis revealed that the uptake of a single violacein-producing bacterium can cause lysis of *A. castellanii* within less than 1 hour. Even though individual cells are ingested and do not survive to reproduce, the remaining clonal prey population may benefit from reduced grazing pressure.

The catastrophic decline in population density suggests the induction of an autolytic process in protozoa feeding on a violacein-producing biofilm. It is known that unicellular eukaryotes, such as protists and fungi, undergo autocatalytic cell death in response to environmental stress, which is analogous to programmed cell death or apoptosis in multicellular organisms [42]. To test whether violacein triggers specifically an apoptosis-like cell death program in protozoan predators, we used morphological and biochemical markers characteristic for apoptosis. Detection of DNA fragmentation by using the TUNEL assay revealed that after 24 hours of incubation 79% of *A. castellanii* cells in the 1 µM violacein treatment and 44% of cells in the 0.1 µM violacein treatment were TUNEL-positive (Fig. 4C and D). Untreated *A. castellanii* remained below 3% while the positive control using 10 µM of the apoptosis inducer actinomycin D had about 17% of positive cells. Simultaneous tests for the maintenance of cell membrane integrity with propidium iodide revealed intact cell membranes despite the degenerative processes inside *A. castellanii* cells (Fig. 4C and D). Analysis with annexin-V antibodies, however, revealed that violacein, similar to actinomycin D, induces the exposure of phosphatidyl serine on the outer layer of the cell membrane in about 20% of *A. castellanii* cells (see Supporting information, Fig. S3). Using colorimetric assays for caspase-like protease activity, we also observed an increase in caspase-3-like activity in *A. castellanii* when exposed to violacein (see Supporting information, Fig. S4). Taken together, these findings suggest that the release of low amounts of violacein from ingested prey bacteria induces an apoptosis-like cell death mechanism in protozoan predators, which not only leads to feeding inactivity but to the effective decline in predator cell densities. Interestingly, violacein isolated from *C. violaceum* has recently been described to induce apoptosis in mammalian cell lines and thus has been proposed as possible novel therapeutic agent for anticancer treatments [43], [44]. Although the molecular target of violacein in the eukaryotic cell remains unidentified, the induction of an apoptosis-like cell death mechanism suggest that an ancient eukaryotic cell process may be the natural target of violacein-mediated defense.

In contrast to predator-prey interactions among plants and animals, the study of antipredator defenses in bacteria is still in its infancy, partly because predation has not been generally recognized by microbiologists as an important fitness determinant for bacteria. However, predation is likely to be particularly intense on “sessile” biofilms, and thus it is something of a conundrum in the microbial sciences how bacteria can build up and persist in such high numbers on surfaces despite the immense consumption rates of their natural predators. One answer to this conundrum is suggested by our results, which introduce chemically mediated resistance to protozoan grazing as a potentially key mechanism underlying grazer resistance in biofilms. We further identify the molecular basis of one biofilm-associated chemical defense, violacein. Assuming that effective chemical defenses are a widespread phenomenon in bacterial communities and generally promoted by the biofilm life form, bacterial antipredator compounds could significantly affect the stability of biofilm communities, the functioning of microbial food webs, and the coupling of energy and nutrient fluxes. Furthermore, we argue that our findings of biofilm chemical defenses may provide a new perspective on the causes of biofilm persistence in infectious diseases as phagocytic predation by protozoa shares fundamental cellular mechanisms with host immune cells.

The long coevolution of bacteria and protists and the considerable metabolic capabilities found in bacteria raise the question of how sophisticated bacteria-protist systems are – an interaction that may have shaped adaptation and diversity at the bacterial-eukaryotic interface more than we currently appreciate.

## Materials and Methods

### Strains and culture conditions

Bacterial strains were isolated from the surface of the green alga *Ulva australis*, collected from the rocky intertidal zone near Sydney, Australia. Unless otherwise stated all bacterial isolates were routinely grown at 25°C on VNSS marine medium [45]. Protozoan predators included common representatives of the three major feeding types: the flagellates *Rhynchomonas nasuta* and *Cafeteria roenbergensis* [26], the ciliates *Tetrahymena* sp. and *Euplotes* sp. (isolated by M. Weitere and V.C.L. Meyer, respectively), and the amoebae *Acanthamoeba castellanii* ATCC 30234 and *A. polyphaga* ATCC 30872. Cultures of *R. nasuta*, *C. roenbergensis*, *Tetrahymena* sp., *A. castellanii* and *A. polyphaga* are axenic and were maintained as described previously [22], [43].

### Grazing bioassay

Biofilm-specific protection against protozoan grazing was tested in a bioassay employing the common marine flagellates, *R. nasuta* and *C. roenbergensis*, as described elsewhere [26]. Bacterial overnight cultures were diluted to 10^5^ cells ml^−1^ in carbon-reduced VNSS medium (0.5 g/l peptone, 0.25 g/l yeast, 0.25 g/l glucose), transferred into 24-well tissue culture plates and grown at 20°C with shaking to give an OD_490 nm_ of 0.2, as determined by a crystal violet staining assay [26]. For the comparison of plankton vs. biofilm persistence, planktonic bacteria were separated from surface-associated cells after 24 h incubation by transferring the planktonic phase of each well to a new plate. Cell numbers of planktonic bacteria were adjusted to a concentration of 1×10^7^ ml^−1^. Subsequently, the suspension-feeder *C. roenbergensis* was added to the planktonic population while the surface-feeder *R. nasuta* was introduced to the biofilm population (both at a final concentration of 1×10^3^ ml^−1^). Numbers of flagellates and planktonic bacteria, and biofilm biomass were followed over four days. In the biofilm assay, surface-associated *R. nasuta* was quantified by inverted confocal microscopy and bacterial biomass by a crystal violet staining assay. In the plankton wells, cell numbers of *C. roenbergensis* and suspended bacteria were determined by epifluorescence microscopy following formalin fixation (2%) and DAPI staining. Generally each treatment was run in replicate wells of four. To examine survival of *Tetrahymena* sp. and *A. castellanii* feeding on violacein-producing biofilms of *P. tunicata*, *P. ulvae*, *P. luteoviolacea*, and *Microbulbifer* sp., experiments were run analogously to the *R. nasuta* design with an inoculum size of 500 amoebae/ml.

### Chemical and genetic analysis of antiprotozoal activity

Freeze-dried cell material of *P. tunicata* was extracted exhaustively with methanol. The active methanol fraction was then partitioned sequentially by flash column chromatography eluting with solvent systems of increasing polarity to yield 29 fractions (hexane, ethyl acetate, dichloromethane, and methanol). These fractions were evaluated for antiprotozoal activity in a 24-well-plate flagellate bioassay, containing *C. roenbergensis* and *R. nasuta* (at a final concentration of 2×10^3^ cells ml^−1^) supplemented with heat-killed *Pseudomonas aeruginosa* prey. Fractions and crude extract were diluted in NSS medium to achieve the desired concentrations in a 500 µl final volume per well. Total flagellate numbers and the number of active cells were monitored by direct inspection with an inverted light microscope and compared to the solvent-only control treatment. The active fractions were further purified by semi-preparative HPLC (Phenomenex, silica gel column, 5 µ, 250×10 mm). Separation was achieved by applying a linear gradient of hexane/ethyl acetate (from 20∶80 to 15∶85) over 35 min. The purified violet pigment was analyzed using a spectrophotometer (DU 640, Beckman), and ^1^H and ^13^C NMR spectroscopy (Bruker DMX 500 MHz). To support the role of violacein in the killing of protozoan predators a *P. tunicata* transposon mutant library was generated using mini-Tn10 transposon [47] and screened for mutants that were defective in violacein production. The DNA sequence of the gene disrupted by the transposon insertion in the violacein negative mutant was obtained using a panhandle PCR method with transposon specific primers, Tn10C (5′-GCTGATTGACGGGACGGCG-3′) and Tn10D (5′-CCTCGAGCAAGACGTTTCCCG-3′) as described [47]. Panhandle PCR products were then sequenced using transposon specific primers (Tn10C and Tn10D) via a primer walking strategy. The DNA sequences were compared to those in the Genbank data-base and additional sequence information was obtained by analysis of the draft genome sequence for *P. tunicata* using a BLAST-search algorithm and gene comparisons made using the integrated microbial genomes system (IMG) [48]. For the specific comparison of *P. tunicata* wild type and *vioA* mutant, biofilms of both strains (OD_490 nm_ = 0.2) were exposed to *R. nasuta* (final concentration of 1×10^3^ ml^−1^) as described above for the grazing bioassay. Numbers of flagellates and biofilm biomass were followed over four days and compared to the non-food control treatment. Each treatment was run in replicate wells of four.

### Violacein production kinetics

Overnight cultures of *P. tunicata*, *P. ulvae*, *P. luteoviolacea*, *Microbulbifer* sp. and *C. violaceum* were diluted to 10^5^ CFU/ml in VNSS medium of four different carbon concentration (100%, 50%, 25%, 12.5%). Biofilms were grown in semi-continuous culture on 24-well microtiter plates for 48 hours. Suspended cells were harvested by centrifugation (14000 rpm, 15 min); washed biofilms were harvested mechanically from the well bottom. Violacein was extracted from cell pellets by a combined treatment of sodium dodecyl sulfate (5% final concentration) and water saturated butanol and quantified photometrically as described elsewhere [49]. Simultaneously, pellets of planktonic and biofilm cells were sampled for the colorimetric quantitation of total protein based on the bicinchoninic acid (BCA) protein assay (Pierce, Rockford, IL). Bacterial overnight cultures were further used for sub-cellular fractionation according to [50].

### Analysis of protozoan fitness

Cultures of *A. castellanii* attached to glass coverslips were exposed to different concentrations of purified violacein (50 µM, 10 µM, 1 µM, 0.1 µM). Numerical and morphological changes in *A. castellanii* populations were monitored over at least 24 hours by determining the number of cells with regular shape ( = active cells) and rounded shape ( = inactive cells). The apoptosis inducer actinomycin D (ICN Biomedicals, Aurora, USA) was used as positive control treatment as described elsewhere [51]. Hallmarks characteristic for eukaryotic cell death programs include DNA fragmentation, cell membrane integrity, phosphatidyl serine exposure and caspase activity [42], [46]. DNA fragmentation was detected by using terminal deoxynucleotidyl transferase-mediated labeling of dUTP nick ends (Roche, Switzerland). The integrity of the amoeba cell membrane was checked by adding propidium iodide. Phosphatidyl serine asymmetry was analyzed by measuring FITC-conjugated annexin V binding to the cell membrane (Sigma, St Louis, USA). The intracellular activity of caspases was determined using the caspase-3 intracellular activity assay kit I (Calbiochem, Germany). All samples were analyzed with the Leica DMLB epifluorescent microscope. A minimum of 100 cells per sample was examined. Experiments were run in triplicate wells and repeated twice.

## Supporting Information

Table S1 (0.05 MB DOC)

Table S2 (0.02 MB DOC)

Table S3 (0.02 MB PDF)

Figure S1 (1.51 MB TIF)

Figure S2 (1.01 MB TIF)

Figure S3 (0.87 MB TIF)

Figure S4 (5.48 MB TIF)
