# Supplementary material for: Marine Biofilm Bacteria Evade Eukaryotic Predation by Targeted Chemical Defense
Source: PLoS One. 2008 Jul 23;3(7):e2744. doi: 10.1371/journal.pone.0002744 (PMC2444038; doi:10.1371/journal.pone.0002744)
Supplement: Table S1 — (0.05 MB DOC) [file pone.0002744.s001.doc]

**Table S1.** Identification of antiprotozoal compounds purified from *Pseudoalteromonas tunicata* D2. NMR data of violacein and deoxyviolacein in *d*6-DMSO were compared with published values from (52).

52. Hoshino T, Kondo T, Uchiyama T & Ogasawara N. (1987) *Agric Biol Chem* 51:965-968.

| ^1^H and ^13^C position | Fraction E | | | | Fraction D | | | |
| --- | --- | --- | --- | --- | --- | --- | --- | --- |
|  | ^1^H NMR | | ^13^C NMR | | ^1^H NMR | | ^13^C NMR | |
|  | Sample | Violacein | Sample | Violacein | Sample | Deoxy-violacein | Sample | Deoxy-violacein |
| 1 | 11.89 | 11.89 | 129.6 | 129.5 | 12.11 | 12.13 | 129.5 | 129.3 |
| 2 | 8.08 | 8.07 | 105.8 | 105.8 | 8.21 | 8.19 | 106.3 | 106.3 |
| 3 |  |  | 125.6 | 125.5 |  |  | 124.5 | 124.4 |
| 4 |  |  | 104.5 | 104.5 |  |  | 120.0 | 119.7 |
| 5 | 7.25 | 7.24 | 152.9 | 152.9 | 7.85 | 7.85 | 123.1 | 123.1 |
| 6 | 9.34 | 9.35 | 113.1 | 113.2 |  |  | 121.6 | 121.5 |
| 7 | 6.79 | 6.78 | 113.4 | 113.4 | 7.31 | 7.30 | 113.0 | 112.9 |
| 8 | 7.35 | 7.35 | 131.6 | 131.5 | 7.56 | 7.56 | 137.5 | 137.4 |
| 9 |  |  |  |  |  |  |  |  |
| 10 | 10.65 | 10.74 | 171.7 | 171.5 | 10.84 | 10.83 | 171.5 | 171.5 |
| 11 |  |  | 137.0 | 136.9 |  |  | 136.8 | 136.7 |
| 12 |  |  | 96.9 | 96.9 |  |  | 97.5 | 97.4 |
| 13 | 7.55 | 7.55 | 147.6 | 147.5 | 7.68 | 7.68 | 147.1 | 147.0 |
| 14 |  |  |  |  |  |  |  |  |
| 15 | 10.74 | 10.64 | 170.2 | 170.1 | 10.65 | 10.67 | 170.1 | 170.1 |
| 16 |  |  | 118.7 | 118.7 |  |  | 119.6 | 119.5 |
| 17 |  |  | 122.4 | 122.3 |  |  | 122.3 | 122.2 |
| 18 |  |  | 126.4 | 126.3 |  |  | 126.3 | 126.4 |
| 19 | 8.93 | 8.93 | 120.8 | 120.8 | 8.95 | 8.96 | 120.9 | 120.9 |
| 20 | 6.95 | 6.95 | 129.4 | 129.3 | 6.96 | 6.96 | 129.7 | 129.7 |
| 21 | 7.20 | 7.20 | 109.0 | 108.9 | 7.22 | 7.22 | 109.0 | 109.1 |
| 22 | 6.82 | 6.82 | 141.8 | 141.8 | 6.83 | 6.83 | 142.0 | 141.9 |
