# Supplementary material for: Marine Biofilm Bacteria Evade Eukaryotic Predation by Targeted Chemical Defense
Source: PLoS One. 2008 Jul 23;3(7):e2744. doi: 10.1371/journal.pone.0002744 (PMC2444038; doi:10.1371/journal.pone.0002744)
Supplement: Table S2 — (0.02 MB DOC) [file pone.0002744.s002.doc]

**Table S2.** Survival of protozoan predators exposed to violacein (10µM) extracted from *P. tunicata* biofilms. Cell counts are pooled from triplicate wells after 24h. Data are means ± SD. Significant differences to the non-food control treatment are indicated (P<0.001).

| Protozoa species | % survivorship |  |
| --- | --- | --- |
|  |  |  |
| Flagellates |  |  |
| Rhynchomonas nasuta | 13 ± 0.5 | *** |
| *Cafeteria roenbergensis* | 7 ± 1.1 | *** |
|  |  |  |
|  |  |  |
|  |  |  |
| Ciliates |  |  |
| *Tetrahymena* sp. | 56 ± 2.9 | *** |
| *Euplotes* sp. | 38 ± 3.4 | *** |
|  |  |  |
|  |  |  |
| Amoebae |  |  |
| Acanthamoeba castellanii | 48 ± 3.6 | *** |
| *Acanthamoeba polyphaga* | 52 ± 4.7 | *** |
|  |  |  |
|  |  |  |
|  |  |  |
