# Supplementary material for: Marine Biofilm Bacteria Evade Eukaryotic Predation by Targeted Chemical Defense
Source: PLoS One. 2008 Jul 23;3(7):e2744. doi: 10.1371/journal.pone.0002744 (PMC2444038; doi:10.1371/journal.pone.0002744)
Supplement: Table S3 — (0.02 MB PDF) [file pone.0002744.s003.pdf]

Table S3. Survival of *R. nasuta*, *Tetrahymena* sp. and *A. castellanii* on violacein-producing biofilms of *P. tunicata, P. ulvae*, *P. luteoviolacea*, and *Microbulbifer* sp. LT_{50} values were used to describe the time needed to kill 50% of the initial predator population. Each value represents the mean ± SD of four replicates.

Bacterial species	LT_{50} of *R. nasuta* (hours)	LT_{50} of *Tetrahymena* sp. (hours)	LT_{50} of *A. castellanii* (hours)
P. tunicata	6 ± 1.0	9 ± 1.8	8 ± 1.2
P. ulvae	15 ± 1.4	18 ± 2.6	17 ± 1.3
P. luteoviolacea	11 ± 1.3	15 ± 2.2	14 ± 1.2
Microbulbifer sp.	2 ± 0.6	5 ± 0.8	2 ± 0.9
