# Supplementary figures and images for: Marine Biofilm Bacteria Evade Eukaryotic Predation by Targeted Chemical Defense
Source: PLoS One. 2008 Jul 23;3(7):e2744. doi: 10.1371/journal.pone.0002744 (PMC2444038; doi:10.1371/journal.pone.0002744)

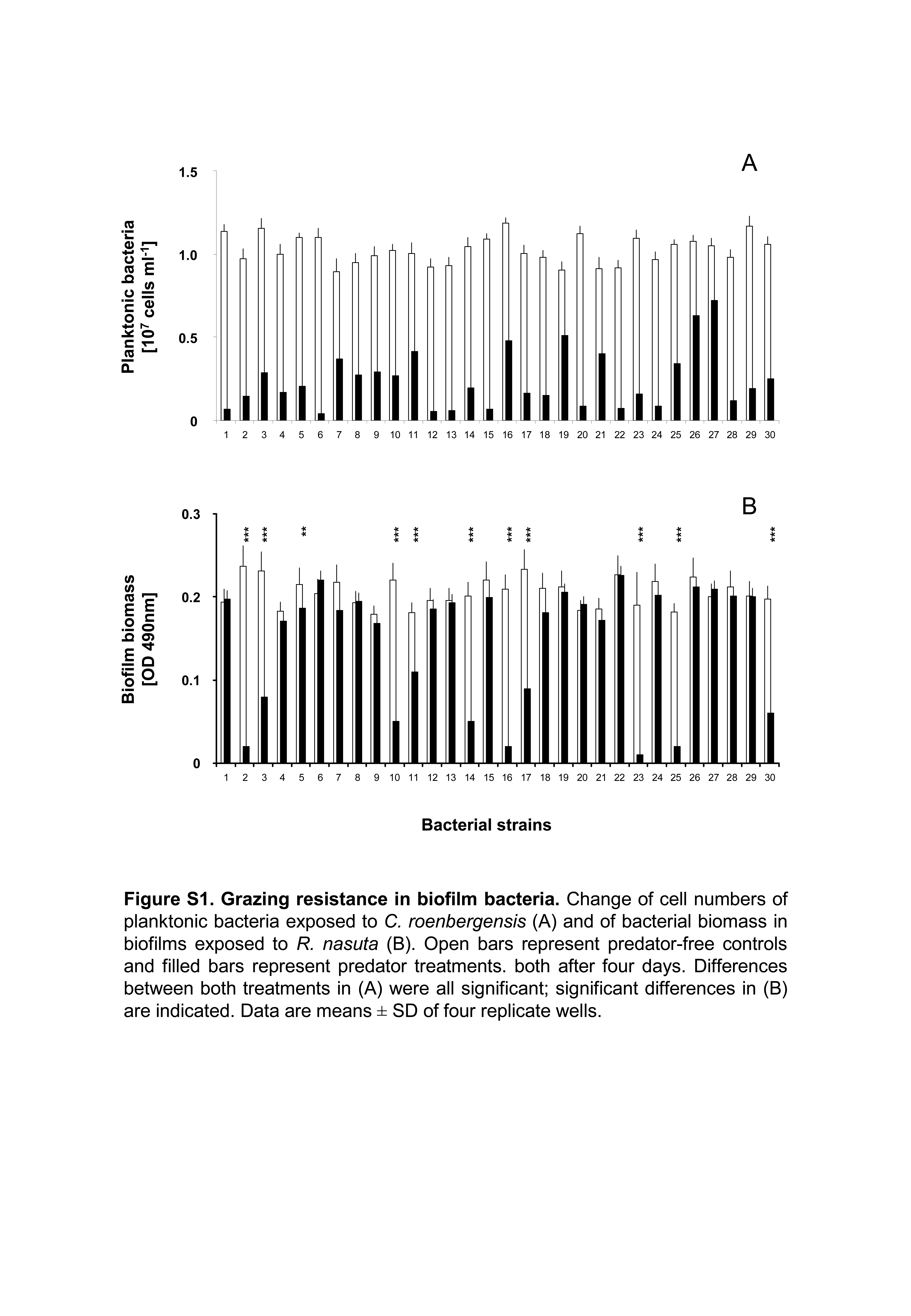

Supplement: Figure S1 — (1.51 MB TIF) [file pone.0002744.s004.tif]

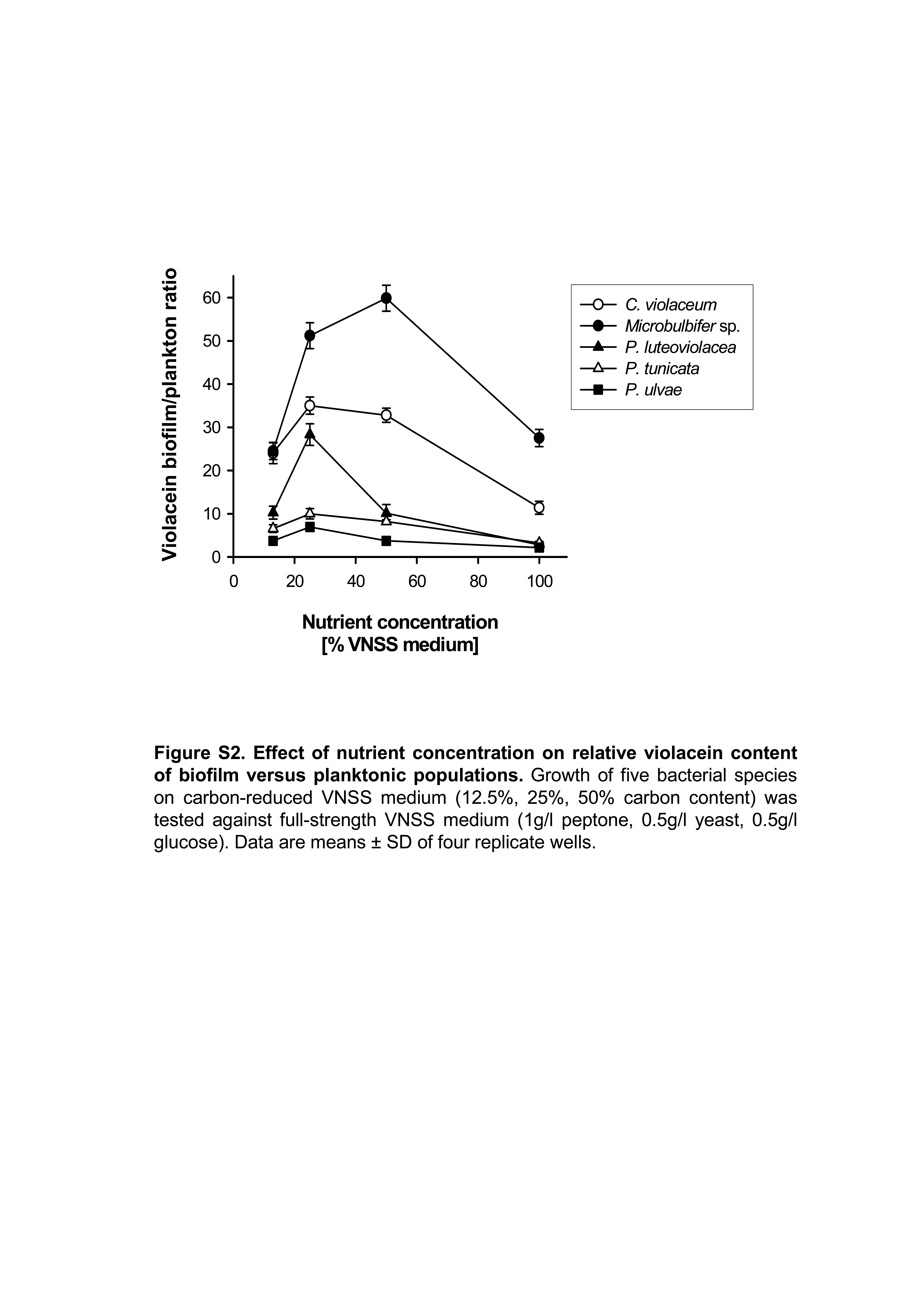

Supplement: Figure S2 — (1.01 MB TIF) [file pone.0002744.s005.tif]

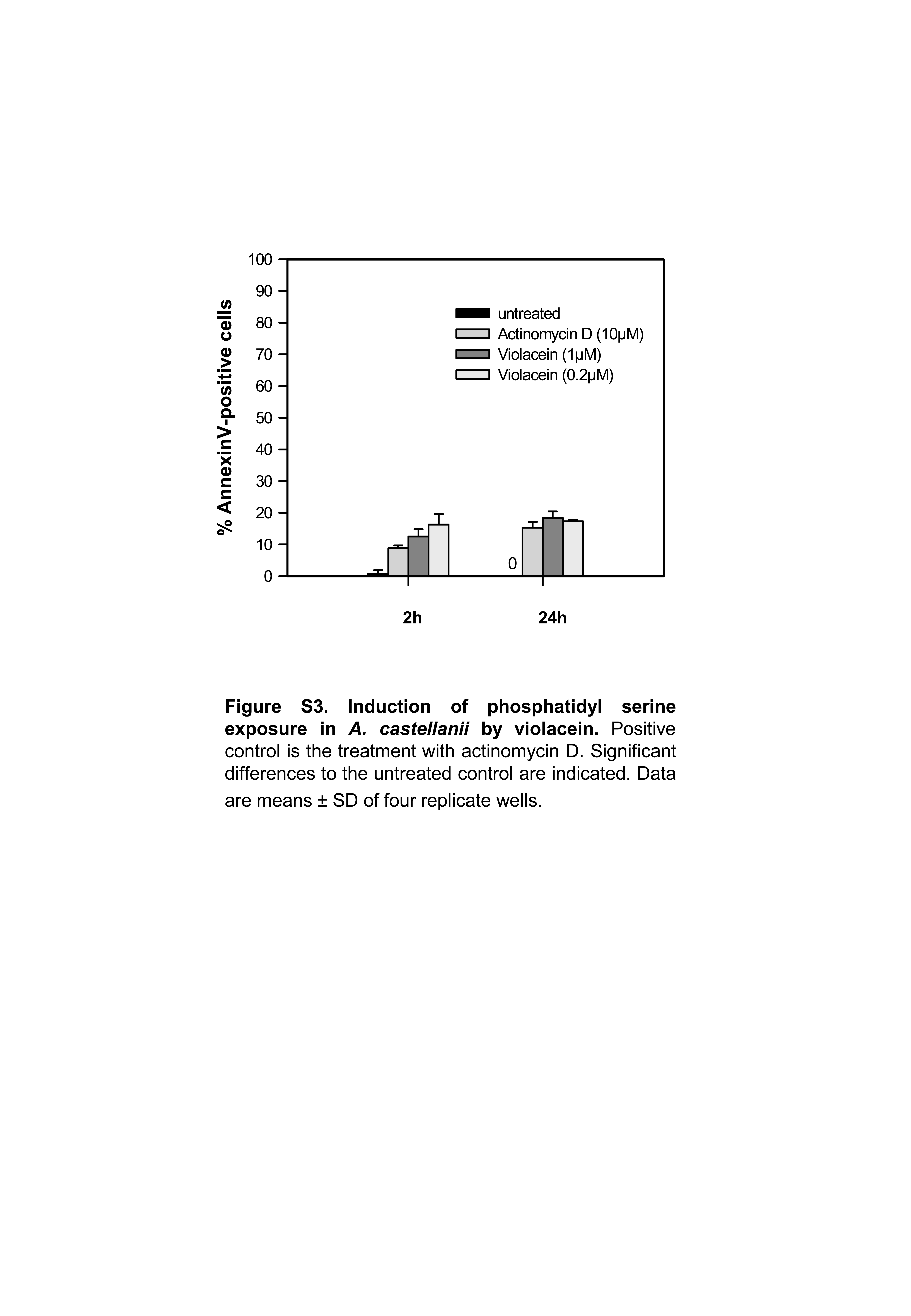

Supplement: Figure S3 — (0.87 MB TIF) [file pone.0002744.s006.tif]

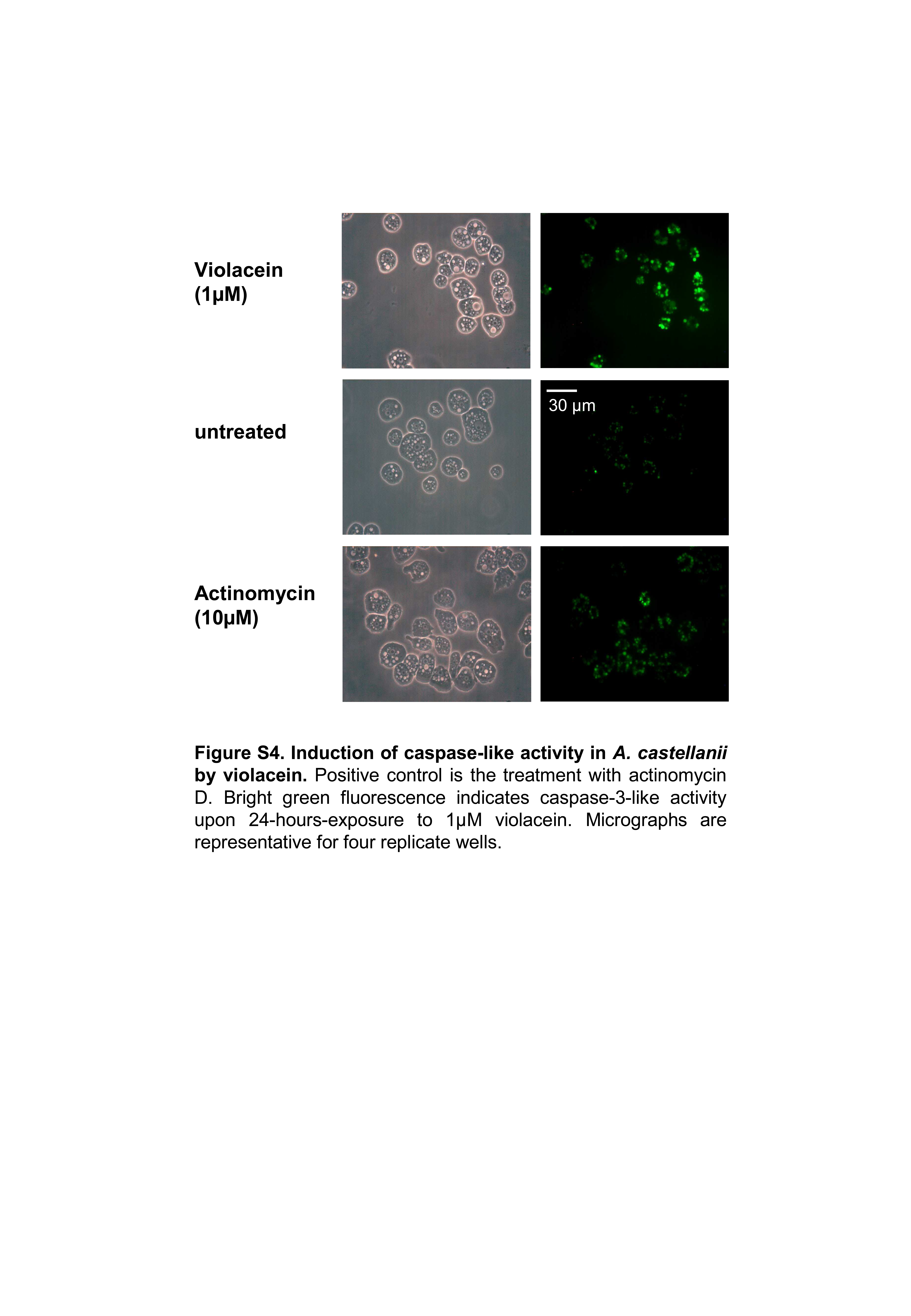

Supplement: Figure S4 — (5.48 MB TIF) [file pone.0002744.s007.tif]
